# Photoelectric Studies as the Key to Understanding the Nonradiative Processes in Chromium Activated NIR Materials

**DOI:** 10.1021/jacs.4c08011

**Published:** 2024-08-02

**Authors:** Natalia Majewska, Mu-Huai Fang, Sebastian Mahlik

**Affiliations:** Ψ Institute of Experimental Physics, Faculty of Mathematics, Physics and Informatics, University of Gdansk, Wita Stwosza 57, 80-308 Gdansk, Poland; † Research Center for Applied Sciences, Academia Sinica, Taipei 11529, Taiwan

## Abstract

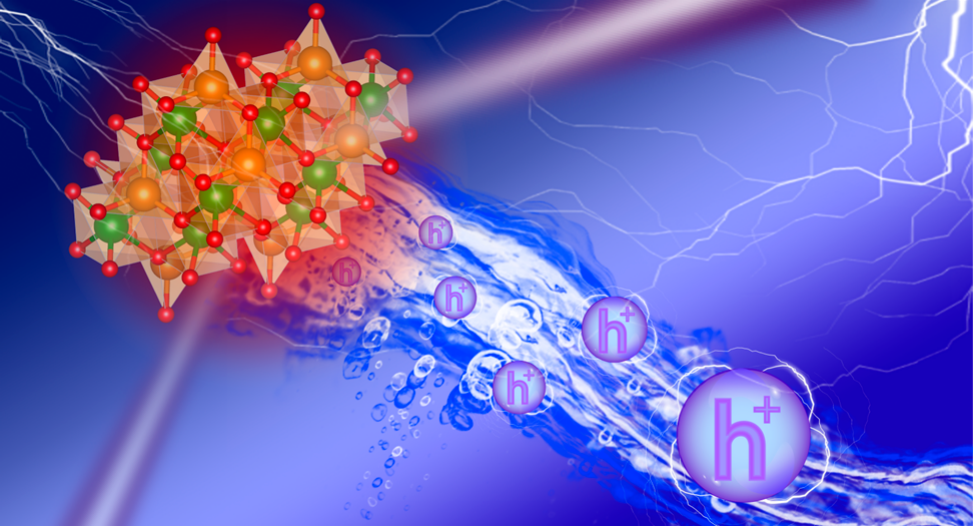

In this study, we synthesized a series of Ga_1.98–*x*_In_*x*_O_3_:0.02Cr^3+^ materials with varying *x* values from 0.0 to 1.0, focusing on their broadband near-infrared emission and photoelectric properties. Interestingly, photocurrent excitation spectra exhibited behavior consistent with the absorption spectra, indicating the promotion of carriers into the band structure by the ^4^T_1_, and ^4^T_2_ states of Cr^3+^ ions. This association suggests that photocurrent in this material is influenced not only by valence to conduction band transitions but also by transitions involving Cr^3+^ dopants. Our investigation of luminescence quenching mechanisms revealed that nonradiative processes were not directly linked to thermally induced relaxation from the excited state ^4^T_2_ to the ground state ^4^A_2_, as usually suggested in the literature for this type of material. Instead, we linked it to the thermal ionization of Cr^3+^ ions. Unexpectedly, this process is unrelated to the transfer of electrons from Cr^3+^ impurities to the conduction band but is associated with the formation of holes in the valence band. This study provided novel evidence of luminescence quenching via the hole-type thermal quenching process in Cr^3+^-doped oxides, suggesting potential applicability to other transition metal ions and host materials. Finally, we demonstrated the dual-purpose nature of Ga_1.98–*x*_In_*x*_O_3_:0.02Cr^3+^ as a practical emitter for NIR-pc-LEDs and effective photocurrent for UV detectors. This versatility underscores these materials’ practicality and broad application potential in optoelectronic devices designed for near-infrared and ultraviolet applications.

## Introduction

The utilization of Cr^3+^-activated optical materials, which exhibit efficient emission within the visible and near-infrared (NIR) spectrum, has garnered significant interest due to emerging demands in fields such as biological imaging, freshness analysis, luminescence thermometry and luminescence manometry exploiting their NIR luminescence capabilities.^1−7^ Particularly, these materials have found practical application in NIR phosphor-converted light-emitting diodes (pc-LEDs), representing a new frontier in NIR light sources owing to their cost-effectiveness, superior efficiency, and compact form factor poised to replace conventional tungsten halogen lamps.^8^ The pc-LEDs leverage high-power blue LED chips combined with inorganic phosphors to achieve targeted NIR emissions.

Despite the extensive research on luminescent materials activated by Cr^3+^ ions documented in the literature, certain fundamental luminescence properties remain inadequately elucidated. Among these is thermal luminescence quenching, a phenomenon observed in all luminescent materials at sufficiently high temperatures, suppressing luminescence. While the radiative processes are well understood, the nonradiative processes and their temperature dependencies, not only for Cr^3+^ ions but also other transition metal ions, are not comprehensively explained. Addressing these nonradiative relaxation processes is pivotal in engineering materials with tailored properties for potential phosphor applications.

According to the models proposed by Struck and Fonger, the activation energy (*E*_nr_) represents the energy required to transition between excited and ground electronic states by the intersystem relaxation.^9^ Despite the widespread acceptance of this approach, these authors have demonstrated that it does not offer an accurate quantitative description of nonradiative quenching processes in materials like Cr^3+^ in ruby and emerald.^10^ The activation energy values observed experimentally for luminescence quenching of transition metal ions such as Cr^3+^ and Mn^4+^ are notably lower by an order of magnitude than predictions from the intersystem relaxation model. Grinberg and Lesniewski have systematically shown that the activation energy for nonradiative transitions (*E*_nr_) for Mn^4+^ ions (which are isoelectronic with Cr^3+^) is significantly smaller than theoretically predicted.^11^ They attribute this discrepancy to the interaction with phonons in a multidimensional configurational space, leading to increased initial state degeneracy. In this context, the final state could be any electronic manifold facilitating nonradiative relaxation, such as an electron trap, impurity-trapped exciton, or conduction band edge. A similar mechanism can be found also in materials activated by Cr^3+^ ions.^12,13^ In certain studies, the activation energy is interpreted as crossing between the ^2^E and ^4^A_2_ levels, despite both belonging to the same electronic configuration and not undergoing such a crossing at all.^14−17^ This highlights the complexity and nuances involved in accurately describing the nonradiative processes governing the luminescent properties of transition metal-activated materials.

Another phenomenon contributing to the reduction of luminescence intensities is autoionization, where an electron in an excited state, influenced by phonon interactions, transitions thermally to the conduction band (CB). In this context, the opposite situation can also be considered, namely the transfer of an electron from the valence band (VB) to the Cr state, causing the formation of intermediate states of the charge transfer (CT) state, known as intervalence charge transfer (IVCT)^18^ or impurity trapped exciton (ITE) state.^19,20^ Instead of electron ionization to the CB, quenching can occur by hole ionization to the VB. These states can significantly affect radiative processes, repeatedly shown in materials doped with, for example, Eu^3+^ ions.^21,22^

The resulting electron in the CB or holes in VB contributes to current carriers, which can be studied through photocurrent measurements. By examining the temperature dependence of photocurrent intensity, we can determine the activation energy of the ionization process. Comparing this with temperature-dependent photoluminescence intensity allows us to establish a link between ionization and luminescence quenching.

Studies on temperature-dependent photoconductivity in dielectric materials activated by transition metal ions, providing insights into luminescence quenching phenomena, remain largely absent in the literature. Some photoconductivity investigations have been conducted on lanthanide ions, particularly Pr^3+^^23−25^ and Ce^3+^ ions,^26−29^ in various matrices. For instance, Tanabe and Ueda observed photoconductivity excitation in Ce^3+^-doped garnets like Y_3_Al_2_Ga_3_O_12_ and Y_3_Ga_5_O_12_, revealing luminescence quenching attributed to thermally activated ionization from the 5*d* excited level, as evidenced by photocurrent excitation spectra.^26,27,30^ They noted that the photocurrent excitation spectrum matches the photoluminescence excitation spectrum at room temperature, indicating that the 5*d*_1_ (lowest excited state) and 5*d*_2_ (higher excited state) states promote carriers into the CB. Concurrently, they observed a decrease in photoluminescence intensity alongside a monotonically increasing photocurrent with rising temperature, demonstrating the role of thermal ionization in the quenching process of Ce^3+^. Similar behavior was observed in other series of Ce^3+^-doped garnets, like Y_3_Sc_2_Al_3–*x*_Ga_*x*_O_12_,^29^ as well as Gd_3_Al_2_Ga_3_O_12_ and Gd_3_Ga_5_O_12_.^28^ While some studies have reported photoconductivity excitation spectra for Cr^3+^-doped LiNbO_3_ materials, which demonstrate photoconductivity associated with Cr^3+^ impurities, these investigations have not yet explored the implications of these findings in relation to luminescence quenching.^31−33^

The Ga_2_O_3_ matrix was chosen as a model sample for investigation for several compelling reasons. First, this material, when codoped with Cr^3+^, exhibits efficient near-infrared luminescence, making it a subject of widespread study as a near-infrared emitter for NIR phosphor-converted light-emitting diodes (pc-LEDs), persistent luminescence phosphors, and optical thermometers. Additionally, Ga_2_O_3_ demonstrates higher current conductivity and photocurrent in the UV region, making it a key candidate for UV detectors, which show numerous applications in space communication, missile and flame warning, or ozone hole detection.^34,35^

Modifying this material with In^3+^ ions allows us to tune the luminescence properties of Cr^3+^ for more suitable performance in NIR-pc-LEDs applications.^36^ Furthermore, this modification can help narrow the band gap, potentially leading to a more significant enhancement of carriers related to the Cr^3+^ ions.^36,37^ This approach holds promise for optimizing the material’s performance and expanding its applicability in optoelectronic devices.

Our research highlights the interplay between photoconductivity and luminescence properties in Cr^3+^-doped materials. Understanding the luminescence quenching mechanisms of materials doped with transition metal ions is crucial for optimizing their luminescence properties for practical applications. This study investigates the luminescence properties of the Ga_1.98–*x*_In_*x*_O_3_:0.02Cr^3+^ (GIOC) solid solution, focusing on its optical and photoelectric characteristics, including the influence of temperature on luminescence and photocurrent. We also demonstrate the potential of Ga_1.78_In_0.2_O_3_:0.02Cr^3+^ as a dual-purpose material, as a potential emitter for NIR-pc-LEDs and UV detectors. This dual functionality showcases the versatility and applicability of this material in optoelectronic devices for both near-infrared and ultraviolet applications. Through this investigation, we aim to pave the way for further advancements in developing efficient and multifunctional materials.

## Results

### Structural Analysis

**Figure 1:**
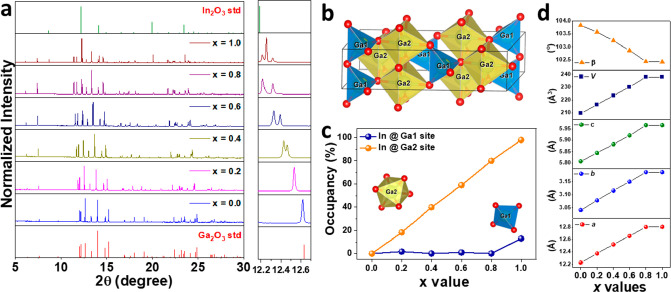
(a) Synchrotron XRD patterns of GIOC (*x* = 0.0–1.0). (b) Crystal structure of Ga_2_O_3_ depicted based on the ICSD-34243. (c) Preferred occupation of In^3+^ at Ga1 and Ga2 sites. (d) Lattice parameters of GIOC (*x* = 0.0–1.0).

To confirm that our experiment successfully synthesized Ga_1.98–*x*_In_*x*_O_3_:0.02Cr^3+^ (GIOC) with *x* = 0.0–1.0, the in-house X-ray diffraction is examined, as shown in Figure S1. The position of the diffraction peak of GIOC fits well with the Ga_2_O_3_ standard pattern converted through the crystallographic information framework (CIF) in inorganic crystal structure database (ICSD) with the number of ICSD-34243.^38^ Besides, the diffraction peaks shift toward the lower angles due to the larger ionic radii of In^3+^ (0.80 Å; CN = 6) (CN represents the coordinated number) than that of Ga^3+^ (0.62 Å; CN = 6).^39^ However, due to the influence of *Kα*2 radiation and the relatively broader peak width of the in-house XRD instrument, the XRD peaks with closed position cannot be well resolved, as denoted by the pound sign in Figure S1. Furthermore, some peaks are connected, hindering the judgment of the contribution from different phases, especially for the *x* = 1.0 sample, as denoted with the asterisk in Figure S1. To address the aforementioned problem, the high-resolution synchrotron XRD patterns of GIOC (*x* = 0.0–1.0) are characterized, as shown in Figure 1a. One can observe that the two diffraction peaks with close positions at 12.6321° and 12.6338° gradually split into two independent peaks. Furthermore, the peaks of *x* = 1.0 at around 12.3° indicate the existence of the two phases, Ga_2_O_3_-phase and In_2_O_3_-phase, proving the benefit of high-resolution synchrotron X-ray diffraction in determining the structural properties. The Ga_2_O_3_ in this study belongs to β-Ga_2_O_3_, possessing a monoclinic structure with a space group of *C*2*/m*, as depicted in Figure 1b. There are two Ga sites, Ga1 and Ga2, in the structure of Ga_2_O_3_. Ga1 is coordinated by four O^2–^ ions, forming a [GaO_4_] tetrahedron, and Ga2 is coordinated by six O^2–^ ions, consisting of a [GaO_6_] octahedron. To further ascertain the structural evolution after incorporating In^3+^ ions, the Rietveld refinement of GIOC (*x* = 0.0–1.0) is conducted, as shown in Figure S2. The atomic positions, occupancies, atomic displacement parameters, and refined parameters are listed in Tables S1–S7. The results indicate that the In^3+^ ions did not distribute evenly at the Ga1 and Ga2 sites. Instead, In^3+^ ions show highly preferred occupation at the Ga2 sites. Only when Ga2 is close to fully occupied by In^3+^ ions (*x* = 1.0), In^3+^ ions will start to incorporate into the Ga1 sites. Besides, the lattice parameters of *a*, *b*, *c* and the volume of the unit cell gradually increase with the increase of In^3+^ ions. In contrast, the β angles of the unit cell are reduced by incorporating In^3+^ ions. Moreover, the XRD pattern of *x* = 1.0 sample can be attributed to the combination of Ga_2_O_3_ phase doped with In and In_2_O_3_ phase doped with Ga, namely Ga_2–*y*_In_*y*_O_3_ (monoclinic, *C*2*/m*) and In_2–*z*_Ga_*z*_O_3_ (cubic, *Ia*3̅), respectively.^40^ The weight ratio between the Ga_2_O_3_ and In_2_O_3_ for *x* = 1.0 sample is around 75%:25%. These results indicate we can only obtain the GIOC in a single phase for *x* = 0.0–0.8. Accordingly, we will use the GIOC (*x* = 0.0–0.8) for the following analysis.

### Room Temperature Photoluminescence Analysis and Energy Gap Determination

**Figure 2:**
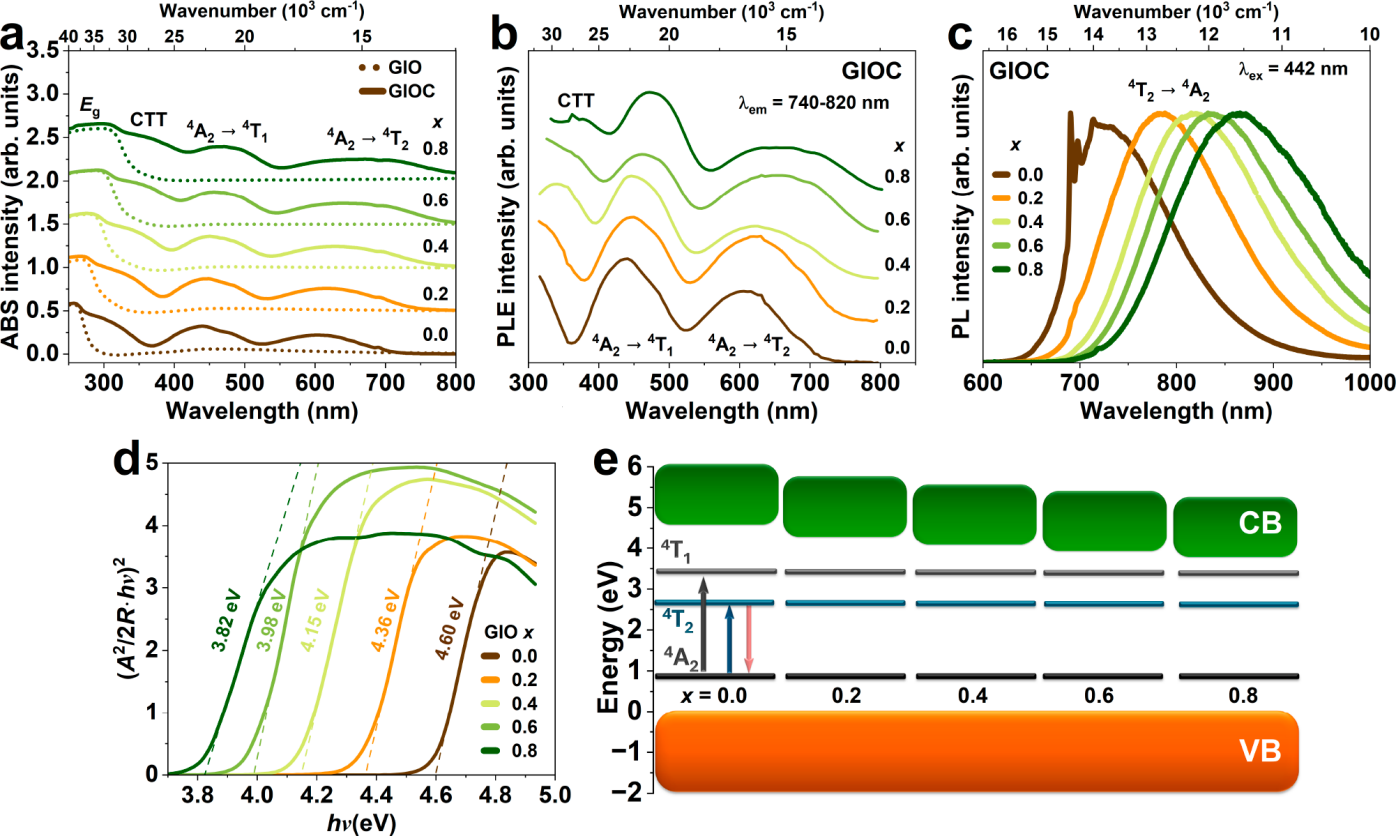
RT (a) ABS spectra of GIOC samples and GIO matrix, (b) PLE spectra upon observation at maximum of luminescence, (c) PL spectra upon excitation at 442 nm. (d) Optical band gap determination for direct band gap. (e) Schematic diagram of energy levels.

To examine the fundamental optical and photoelectrical properties, room temperature (RT) absorption (ABS), photoluminescence excitation (PLE) and photoluminescence (PL) spectra of GIOC for *x* = 0.0–0.8 are shown in Figure 2. The ABS spectra reveal one overlapping band in the ultraviolet (UV) and two distinct bands in the visible (VIS) region. The absorption spectra of the undoped samples Ga_2–*x*_In_*x*_O_3_ (GIO, crystal matrix) are depicted in Figure 2a with dashed lines, showing a single band in the UV region. This indicates that the UV bands observed in GIO and GIOC are associated with band-to-band (VB → CB) absorption with the energy related to the band gap (*E*_g_). In contrast, the lower energy bands observed solely in GIOC are attributed to Cr^3+^ dopants. Specifically, the 300 nm band for *x* = 0.0 could be attributed to the charge transfer transition (CTT).^41^ The ∼450 nm and ∼620 nm bands correspond to the ^4^A_2_ → ^4^T_1_ and ^4^A_2_ → ^4^T_2_ transitions of Cr^3+^ ions in a 6-fold octahedral coordination. All bands exhibit a wavelength red shift (shift toward lower energies) with increasing *x*. This red shift is attributed to the decreased crystal field strength (*Dq*) around Cr^3+^ ions due to substituting larger In^3+^ ions for Ga^3+^ ions, increasing in lattice volume, as evidenced by XRD studies in Figure 1. The decrease in *Dq* value lowers the energy levels of the ^4^T_1_ and ^4^T_2_ states relative to the ^4^A_2_ ground state.

Upon observation at the maximum luminescence of GIOC (740–820 nm), the PLE spectra reveal three excitation bands of Cr^3+^ ions, consistent with the ABS spectra (Figure 2b). Furthermore, the lower energy excited band associated with the ^4^A_2_ → ^4^T_2_ transition exhibits significant broadening with increasing *x*. This broadening can be attributed to the increased disorder or variability in the local crystal field environment surrounding the Cr^3+^ ions as the concentration of *x* increases.

For *x* = 0.0, both narrow-line and broadband emissions of Cr^3+^ are observed simultaneously (Figure 1c). The narrow-line emission around 690 nm corresponds to the ^2^E → ^4^A_2_ transition (R_1_ and R_2_ lines), accompanied by a weak phonon structure. The presence of the line emission suggests that the *x* = 0.0 sample exhibits a strong crystal field environment. The line emission is visible only for samples with low In^3+^ concentration, specifically *x* = 0.0 and 0.2. The broadband emission from 650 to 950 nm corresponds to the transition from the ^4^T_2_ excited to the ^4^A_2_ ground state. Co-doping with In^3+^ induces a red shift in the emission spectra related to the ^4^T_2_ → ^4^A_2_ transition due to the decreasing *Dq* value.

At RT, the full width at half-maximum (*fwhm*) for *x* = 0.0 is 2064 cm^–1^. Upon incorporating In^3+^, the *fwhm* increases significantly to 2351 cm^–1^ for *x* = 0.2 and then slightly increases up to 2385 cm^–1^ for *x* = 0.8. Similarly, *S*ℏω values, which describe the energy of electron lattice relaxation, significantly increase from 1415 cm^–1^ (*x* = 0.0) to 1639 cm^–1^ (*x* = 0.2) and then slightly increase up to 1654 cm^–1^ for *x* = 0.8. As expected, the crystal field strength (*Dq*) decreases with increasing *x* value, from 1647 to 1490 cm^–1^. The increasing *fwhm* and *S*ℏω values are attributed to the enhanced disorder in the environment surrounding the Cr^3+^ centers, induced by the incorporation of In^3+^ into the crystal lattice. Detailed analysis of these parameters is provided in the Supporting Information (SI) in Table S8.

Figure S3a displays the RT concentration-dependent decay profiles upon excitation at 440 nm. The decay profile is single-exponential for the sample without In^3+^ dopants (*x* = 0.0), whereas In^3+^-doped samples exhibit increasingly multiexponential decay behavior, due to aforementioned variations of the local surroundings. The average decay time (τ_av_) for all studied materials was calculated using the following eq 1:
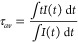
1where *I*(*t*) is the luminescence decay profile. The calculated average decay times of Cr^3+^ ions are illustrated in Figure S3b. The incorporation of In^3+^ ions into the crystal structure of Ga_2_O_3_:Cr^3+^ results in an extended crystal lattice, which reduces the crystal field strength around the Cr^3+^ ion. Consequently, as the *x* increases, there is an increase in the thermal occupation of the ^4^T_2_ state, resulting in two observable effects: increasing prominence of the ^4^T_2_ → ^4^A_2_ broadband emission and shortening of the emission decay time. This latter effect is demonstrated in the RT luminescence decay time study shown in Figure S3a and S3b.

The optical band gap energy (*E*_g_) of the studied samples can be estimated for each sample from the diffuse reflectance spectra (Figure 2d) using the following eq 2:^42^
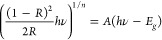
2where *hv*, *R*, *E*_g_, and *A* refer to the photon energy, reflectance, band gap, and proportionality constant, respectively. The value of the exponent *n* in the equation used to estimate the nature of the electronic band and transition type determines the optical band gap. For materials with direct band gaps, *n* typically has a value of 1/2, while for those with indirect band gaps, *n* is 2.

In β-Ga_2_O_3_, there is an indirect band gap, with the direct band gap only slightly larger by approximately 0.04 eV. The direct transitions have a higher probability, so we assume that these transitions are observed in the absorption spectra. Therefore, we used *n* = 1/2 for the direct band gap calculations,^43^ resulting in the *E*_g_ = 4.60 eV for *x* = 0.0, which are slightly smaller than those reported in refs (36 and 44−46), *E*_g_ = 4.69–4.8 eV. The band gap gradually narrows with increasing *x*, with determined values of 4.36, 3.15, 3.98, and 3.82 eV for GIO (*x* = 0.2–0.8), respectively (Figure 2d). As demonstrated by He et al., the band gap of β-In_2*z*_Ga_2(1–*z*)_O_3_ decreases linearly with increasing In^3+^ concentration, attributed to the rising valence-band maximum and the lowering conduction-band minimum.^37^ The band gap values estimated for the indirect transitions are shown in Figure S3c. These values are significantly lower than those reported in the literature.^36,44−46^Figure 2e shows the schematic diagram of energy levels showing the decrease of the *E*_g_, and the Cr^3+^ dopant states. The energy levels of the Cr^3+^ dopants were taken from the ABS/PLE spectra, and energy equal to 0 was fixed at the edge of the VB.

### Room Temperature Photocurrent Analysis

**Figure 3:**
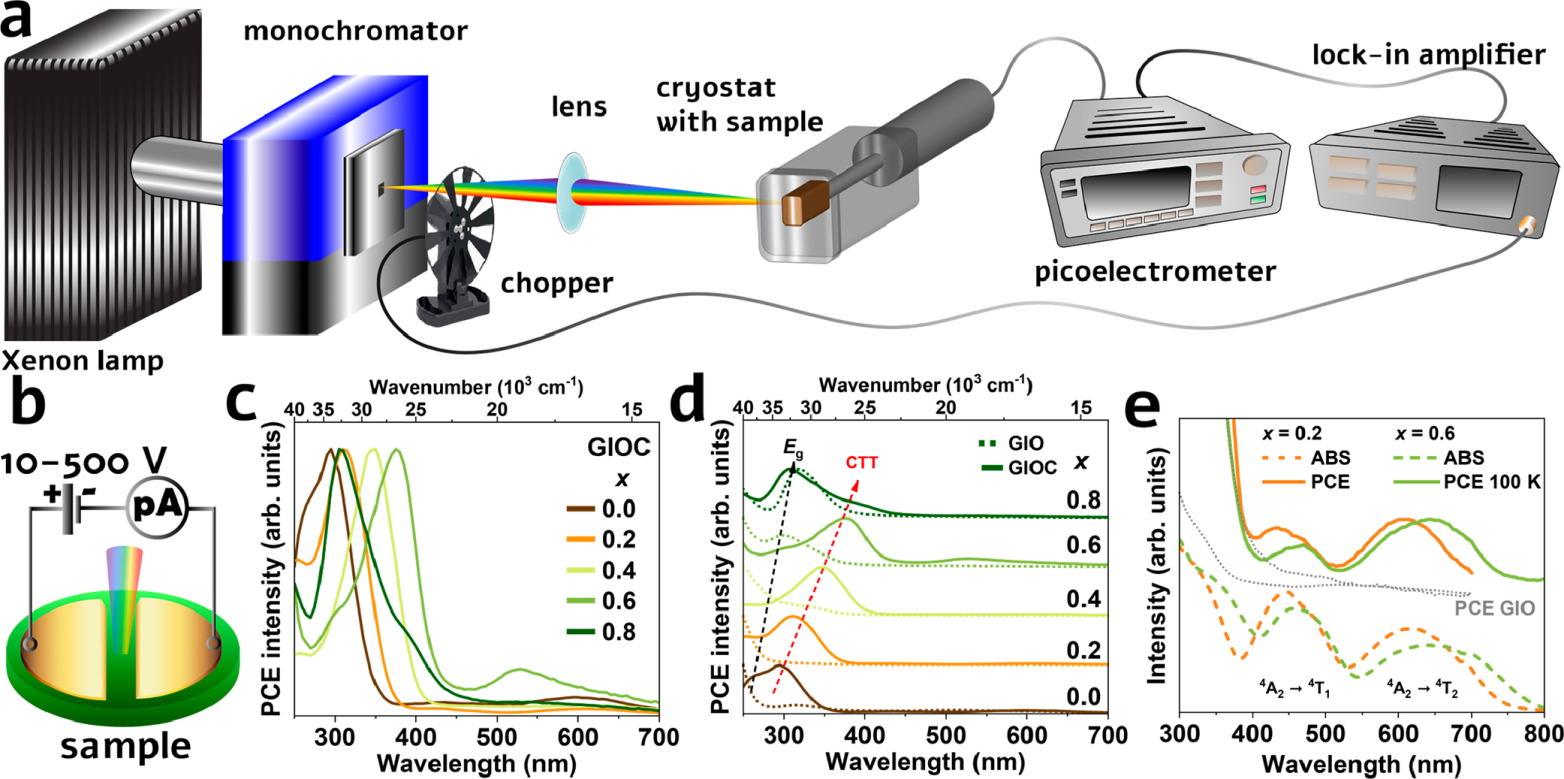
(a) Schematic photocurrent excitation measurement system. (b) Schematic view of the sample preparation. RT PCE spectra of (c) GIOC samples and (d) GIOC samples compared with GIO matrix as a reference. (e) Comparison of PCE and ABS of GIOC *x* = 0.2 and 0.8.

Figure 3a illustrates the schematic diagram of the photocurrent excitation measurement system used in the study. The setup begins with a xenon lamp emitting a broad-spectrum light beam that passes through a grating monochromator, which selects a specific 10 nm *fwhm* section of the lamp spectrum. This monochromatic beam is then directed through a focusing lens onto the sample, where two gold electrodes, separated by approximately 200 μm, are positioned (Figure 3b). The size of the light spot from the monochromator determines the separation distance between the gold electrodes. The gold electrodes, shaped like semicircles and deposited through a shadow mask using magnetron sputtering, have a thickness of approximately 100 nm. Due to the persistent photocurrent observed in the studied material and the associated charging and discharging processes,^47−49^ a lock-in amplifier combined with chopper modulation is employed to isolate the photocurrent signal from background noise. This setup allows for accurate measurement of the photocurrent response.

Figure 3c displays the normalized photocurrent excitation (PCE) spectra of GIOC for *x* = 0.0–0.8. The applied voltage was 500, 300, 200, 20, and 10 V, for *x* = 0.0–0.8, respectively. These applied voltages are adjusted to optimize measurement conditions and ensure the dark current in the pA range across the sample series. For *x* = 0.0, a prominent band is observed in the UV region at 300 nm in the PCE spectra. Additionally, less intense bands in the visible (VIS) range are observed at 440 and 610 nm. As *x* increases, the UV band red shifts. Similarly, the VIS bands also red shift with increasing *x*. However, for *x* ≥ 0.4, the spectra exhibit changes where an additional band at 500 nm appears. Further temperature dependence studies reveal that this additional band, appearing for samples *x* ≥ 0.4, covers the observed two VIS bands, still clearly resolved at lower temperatures. Comparatively, the PCE spectra for the GIO (undoped matrix) are shown in Figure 3d (dashed line) alongside the PCE of GIOC. Notably, the band at ∼300–400 nm observed in GIOC is absent in the PCE spectra of the GIO matrix. This indicates that the PCE in the GIOC samples is associated with band-to-band transitions within the matrix together with transitions involving Cr^3+^ dopants. The UV band in the PCE spectra consists mainly of the band-to-band transition (*E*_g_) at 260 nm and the CTT from the VB to Cr^3+^ state (as further analyses prove) at 300 nm for *x* = 0.0 in GIOC. The *E*_g_ and CCT bands red shift with increasing *x*. For *x* = 0.8, the CCT band decreases significantly, and the *E*_g_ band dominates the PCE spectra. Comparison of the ABS and PCE spectra of GIOC for *x* = 0.2 and 0.6 (Figure 3e) indicates that the PCE bands in the visible range align with the ABS bands. Both ABS and PLE (photoluminescence excitation) spectra of *x* = 0.6 show red shifts compared to *x* = 0.2. Furthermore, the lower energy band around 650 nm is broadened for both PCE and ABS spectra. Notably, these bands are not observed in samples without Cr^3+^ (GIO matrix), highlighting their association with the ^4^A_2_ → ^4^T_1_ and ^4^A_2_ → ^4^T_2_ transitions of Cr^3+^. It should be noted this study represents the first observation of photocurrent related to the Cr^3+^ doped dielectric lattice.

### Temperature-Dependent Photoluminescence and Photocurrent Analysis

**Figure 4:**
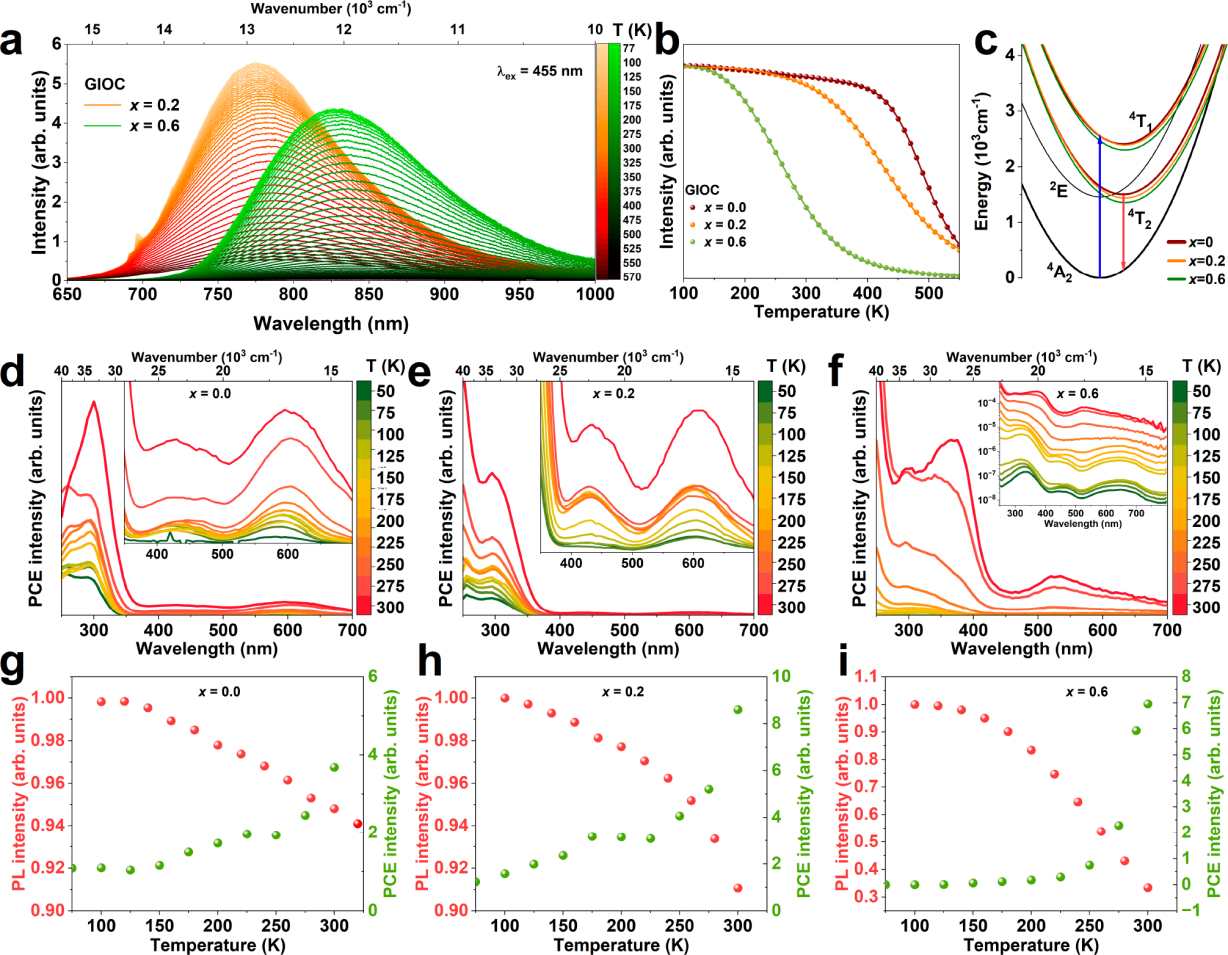
Temperature-dependent (a) emission spectra of GIOC, *x* = 0.2, and 0.6, and (b) integrated PL intensity of GIOC, *x* = 0.0, 0.2, and 0.6 sample. (c) Configuration diagram of GIOC *x* = 0.0, *x* = 0.2, and *x* = 0.6. The temperature dependence of PCE spectra of GIOC (d) *x* = 0.0, (e) *x* = 0.2, and (f) *x* = 0.6. Comparison of integrated PL and PCE intensities of (g) *x* = 0.0, (h) *x* = 0.2, and (i) *x* = 0.6.

PL and PCE measurements were conducted while varying the temperature to enhance understanding of radiative and nonradiative processes, focusing specifically on samples *x* = 0.0, 0.2, and 0.6. Figure 4a shows the temperature-dependent PL spectra in the temperature range of 77–550 K for *x* = 0.2 and 0.6 upon excitation at 455 nm. The temperature-dependent PL spectra of *x* = 0.0 are shown in Figure S4. Given the widely reported temperature behavior of emission spectra in Ga_2_O_3_:Cr^3+^ systems, we will not focus on the temperature-induced changes in emission spectra for the *x* = 0.0 sample.^50−52^ However, it is essential to note that the broadband ^4^T_2_ → ^4^A_2_ emission, extending from 650 to 1000 nm, is observed for all samples in the tested temperature range, with the emission broadening observed with increasing temperature. In the case of the *x* = 0.2 sample, additional emission lines around 700 nm related to the ^2^E → ^4^A_2_ transition are also observed. This can be attributed to the distribution of Cr^3+^ local environments and the crystal field strength, which, in the case of the *x* = 0.2 sample, spans across the crossing point of the ^2^E and ^4^T_2_ states for luminescent centers. This phenomenon has been described in similar materials in refs (53 and 54).

Figure 4b illustrates the temperature-dependent emission intensity of Cr^3+^ for samples *x* = 0.0, 0.2, and 0.6. In the case of the *x* = 0.0 sample, the material exhibits excellent thermal stability, with only a gentle decrease in emission intensity of about 10% observed up to 400 K. However, a significant reduction in emission intensity occurs at higher temperatures. For samples doped with In^3+^, the considerable decrease in emission intensity starts at lower temperatures. Specifically, for *x* = 0.2, the emission intensity decreases significantly around 250 K, while for *x* = 0.6, the decrease starts at 150 K. The experimental data on the temperature dependence of Cr^3+^ emission intensity can be adequately fitted using a formula (3) for two deexcitation processes, reflecting the complex thermal behavior observed in these materials.
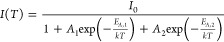
3where *I*_0_ is the PL intensity at 77 K, *E*_A,1_, *E*_A,2_ are the activation energy, and *A*_1_ and *A*_2_ are the relative probability of the nonradiative deexcitation processes. The fitting curves represented by solid lines in Figure 4b correspond to the obtained parameters listed in Table 1.

**Table 1:** Parameters Obtained from Fitting the Temperature-Dependent Integrated Emission Intensity

	***x*** **= 0.0**	***x*** **= 0.2**	***x*** **= 0.6**
***E***_**A,1**_**(cm**^**–1**^**)**	450 ± 10	930 ± 50	760 ± 9
***A***_**1**_	0.5 ± 0.2	9 ± 2	43 ± 3
***E***_**A,2**_**(cm**^**–1**^**)**	5460 ± 30	3270 ± 80	2020 ± 20
***A***_**2**_	(7.3 ± 5.4) × 10^6^	(3.3 ± 0.7) × 10^4^	(1.2 ± 0.1) × 10^4^

The activation energy *E*_A,1_ is relatively low; moreover, the probability of this process is negligible, as evidenced by the *A*_1_ parameter, which is several orders of magnitude lower than the A_2_ parameter describing the following quenching mechanism. This process may be related to the tunneling effect to the ground state ^4^A_2_ from the excited ^4^T_2_ after excitation to the excited vibronic states in Cr^3+^ centers.

The activation energy *E*_A,2_ corresponds to the nonradiative thermal quenching of Cr^3+^ luminescence, which can be attributed to three primary mechanisms:

(i) Photoionization: This mechanism involves the transfer of the excited electron from the ^4^T_2_ state of Cr^3+^ to the CB due to thermal energy.
(ii) Crossover mechanism: In this process, thermal activation energy associated with the intersection of the excited and ground states allows electrons to relax directly from the ^4^T_2_ to the ^4^A_2_ ground state.
(iii) Electron transfer from the ^4^T_2_ state of Cr^3+^ to the CT(ITE) state (Cr^3+^+e).

The Stokes shift between excitation and emission spectra allowed us to construct one-dimensional configurational coordinate diagrams, which consist of the ground state (represented by a black parabola) and excited states (represented by colored parabolas) of Cr^3+^ for *x* = 0.0, 0.2, and 0.6 (Figure 4c). By analyzing these configuration diagrams, we can conclude that the nonradiative processes are not solely attributed to the thermally induced nonradiative relaxation process directly from the excited state to the ground state (^4^T_2_ → ^4^A_2_ transition). The intersections of the ground and excited state parabolas occur at much higher energies for Cr^3+^ (∼20 000 cm^–1^), suggesting that additional processes or energy levels are involved in the nonradiative relaxation pathways of the Cr^3+^ ions. For further insights into the temperature dependence of luminescence kinetics, refer to the discussion in the SI, specifically Figure S5.

To assess whether the ionization process may cause luminescence quenching, we analyzed the temperature dependence of PCE spectra for GIOC samples *x* = 0.0, 0.2, and 0.6, Figure 4d, e, and f, respectively, covering a temperature range of 50–300 K. Insets in the figures show a smaller PCE range to highlight less intense bands in the visible range. Notably, for *x* = 0.6, the inset is shown in the log scale due to the stronger temperature dependence observed in this case. As depicted in Figure 4d, e, and f, the overall PCE increases with rising temperature. Visible range bands related to the ^4^A_2_ → ^4^T_1_ and ^4^A_2_ → ^4^T_2_ transitions of Cr^3+^ are also observed at low temperatures. Integrated PCE intensities are presented in Figure 4f on a log scale, revealing a significant increase for the *x* = 0.6 sample compared to *x* = 0.0 and 0.2.

Comparing the integrated PCE intensity with PL intensity for samples *x* = 0.0, 0.2, and 0.6 (Figures 4g, h, i, respectively), we note a slight decrease in PL intensity up to 300 K for *x* = 0.0 and 0.2, accompanied by a slight increase in PCE intensity. Conversely, for *x* = 0.6, a significant decrease in PL intensity aligns with a growth of photocurrent. This suggests that the decreasing PL intensity is related to the promotion of carriers to the bands. These results prove that luminescence quenching in these materials is linked to ionization. The observed photocurrent in which the Cr^3+^ excitation bands are visible even at low temperatures would suggest that the ^4^T_2_ state lies very close to the CB edge, allowing for the thermal activation of electrons from ^4^T_2_ to the CB. In such a case, the higher excited state ^4^T_1_ of Cr^3+^ ions would degenerate with the CB. This means that excitation to higher states would give a higher photocurrent, and other temperature dependences of the photocurrent would be expected when excited to the ^4^T_2_ and ^4^T_1_. No such effect was observed; moreover, considering previous papers showed significant distance between the ^4^T_2_ state and CB edge, this mechanism of thermal excitation of electrons from Cr^3+^ into the CB should be excluded.^36^ The observed photocurrent should therefore be considered as the transport of electric charges in VB namely a hole current.

### Model

**Figure 5:**
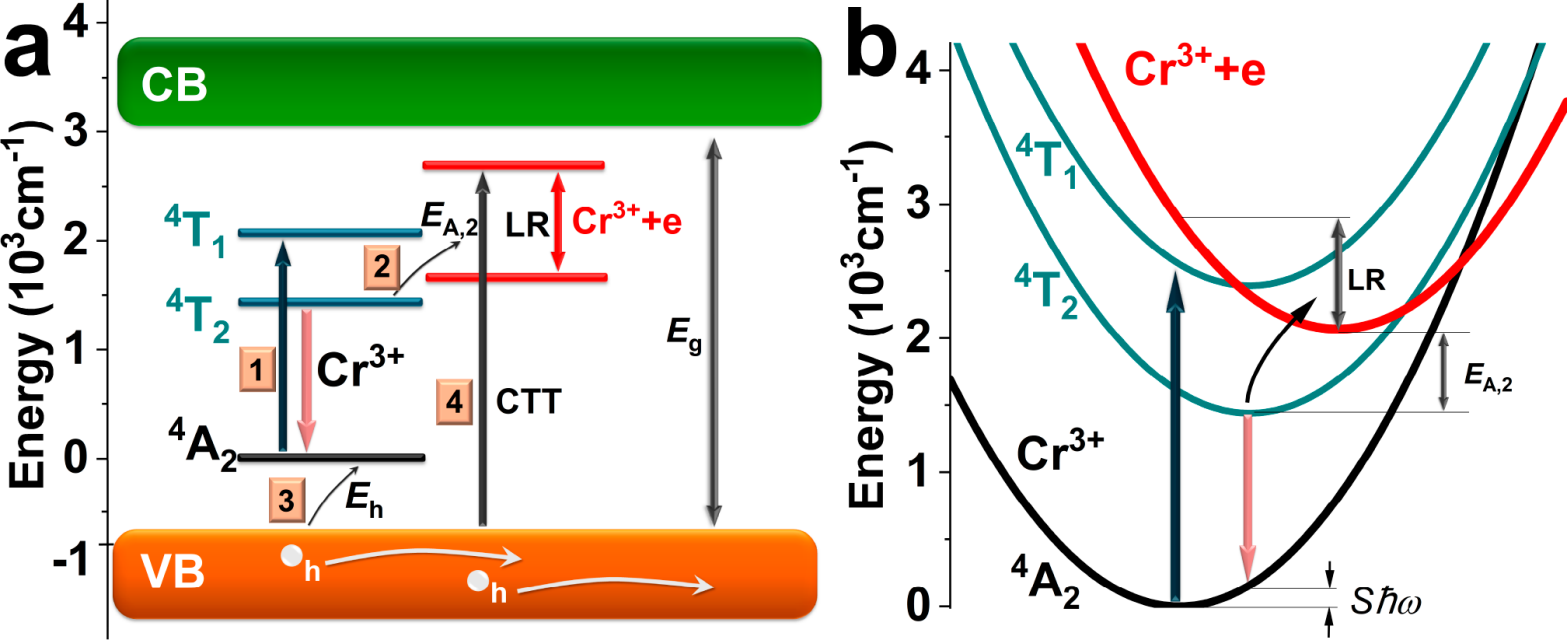
Energy diagram describing the kinetics of the observed processes. (b) Configuration diagram for the Cr^3+^ ion, including the CT state (Cr^3+^+e).

The kinetics of the processes occurring in the tested systems are presented in Figure 5a. Energy zero was set at the basic level of the Cr^3+^ ion. The *E*_g_ defines the width of the energy band gap that changes for the sample series as presented in Figure 2. Material *x* = 0.2 was chosen as an example; however, the process described applies to all materials tested. Selected levels of the Cr^3+^ ion and the CT state (in which the additional electron is trapped on the Cr^3+^ ion) are marked in the energy gap. The position of the Cr^3+^ states in relation to the band edges was taken from ref (36). When the Cr ion captures electrons (Cr^3+^ + e), strong lattice relaxation (LR) occurs, which is illustrated by lowering the energy of the CT state in the diagrams. This situation is better illustrated by the configuration diagram in Figure 5b, in which a red parabola describes the CT state. The position of the CT state (Cr^3+^ + e) was determined considering the CTT transition and the *E*_A,2_ activation energy, which allowed us to determine the lattice relaxation energy LR.

The ground state of the Cr^3+^ ion is close to the VB edge, which may favor the transfer of charges from the VB to the Cr^3+^ ion states. At the same time, the excited ^4^T_2_ state is away from the CB edge, which prevents electrons thermal ionization into the CB. Upon excitation of the Cr^3+^ ion (1), a fast nonradiative relaxation to the ^4^T_2_ excited state occurs. From the ^4^T_2_ state, we can observe broadband emission (bright pink arrow), which is extinguished as the temperature increases. This quenching occurs due to electron transfer to the CT (2) state, which is distant from the ^4^T_2_ state by the activation energy *E*_A,2_. The proximity of the ground state of the Cr^3+^ ion to the VB edge allows for electron capture by the Cr^2+^ ion (Cr^3+^ – e), which gave up an electron to the CT state, leaving a hole in the VB (3). We can also observe the creation of holes by directly exciting electrons from VB to the CT state (4). Such CTT is clearly visible in the photocurrent excitation spectra (see Figure 3b). The electron from the CT state can be transferred back to the Cr ions. Still, this process can only occur if the Cr^3+^ ion has not captured the electron from the VB. All this causes the CT to become a metastable state, and persistent luminescence^55^ and photocurrent are observed in the tested systems. It should be noted that the electron current observed in undoped materials is strongly quenched by the CT states (which are deep electron traps).

### One Sample-Two Applications

Finally, we present a dual-purpose material, GIOC, highlighting its suitability as both an emitter for NIR-pc-LEDs and a UV detector. This dual functionality underscores the versatility and practicality of this material in optoelectronic devices designed for applications in both the near-infrared and ultraviolet regions.

**Figure 6:**
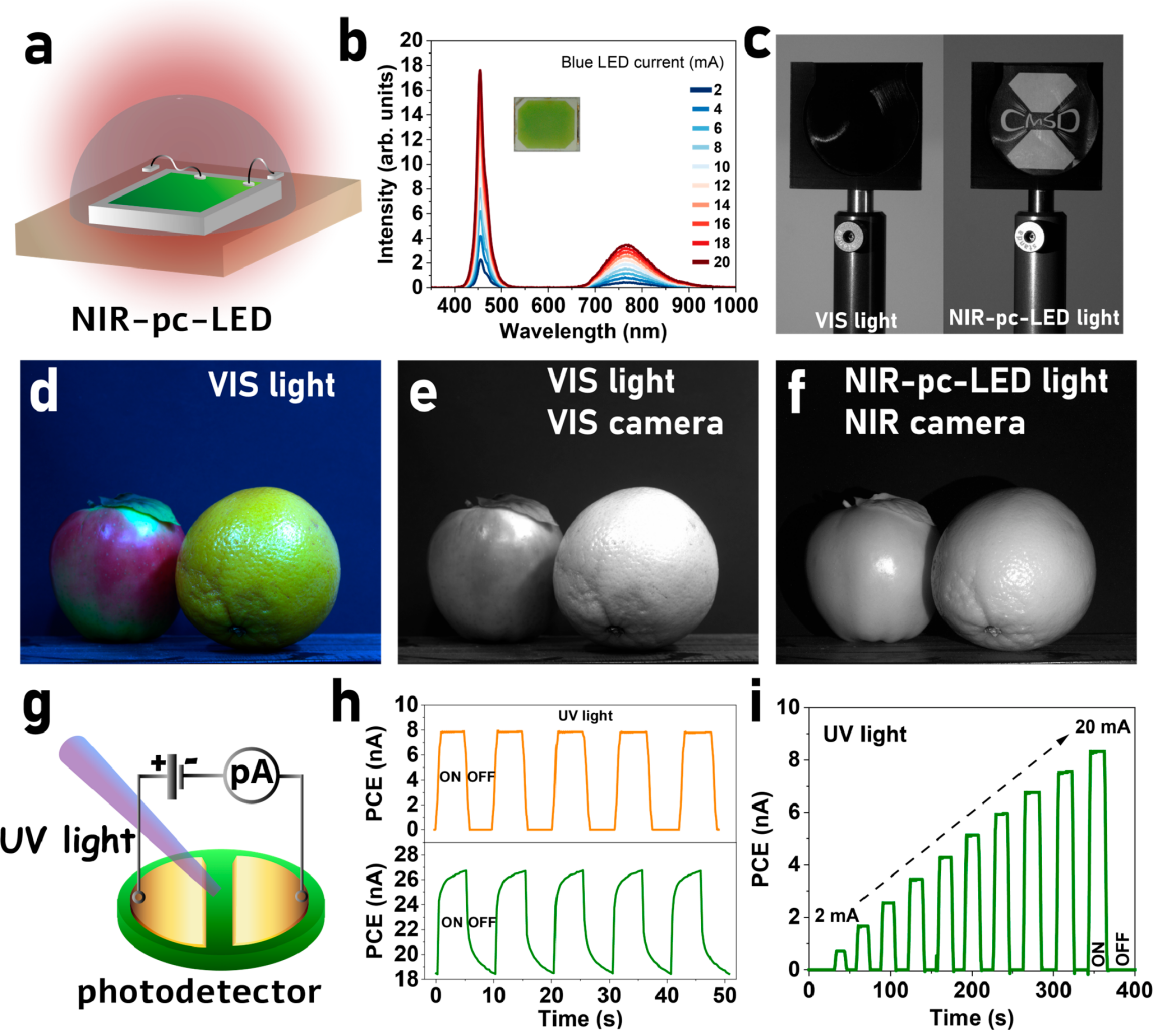
(a) Schematic illustration of NIR-pc-LED. (b) Photoluminescence spectrum of the fabricated NIR-pc-LED device that combines a 450 nm InGaN blue LED chip with Ga_1.78_In_0.2_O_3_:0.02Cr^3+^ NIR phosphor under a forward bias of 2–20 mA, and the insets show the photographs of the LED device. Photographs obtained under VIS light and pc-NIR-LED captured by the corresponding VIS camera and a NIR camera, showing (c) that the produced NIR light passes through the 800 nm long pass filter and (d–f) the obtained images. (g) Schematic illustration of fabricated UV detector. (h) Photocurrent measured at 20 V and under 290 nm UV-LED excitation. (g) Dependence of photocurrent on applied excitation source UV-LED current.

The *x* = 0.2 sample was selected for NIR-pc-LED preparation due to its highest intensity upon a blue 450 nm LED excitation (see Figure S7a). Figure 6a illustrates the schematic of the NIR-pc-LED package. The fabricated LED device was driven by currents ranging from 2 to 20 mA, resulting in an emission peak centered at 770 nm with *fwhm* of 115 nm (Figure 6b). As depicted in Figure 6b, increasing the current from 2 to 20 mA led to a gradual increase in NIR intensity. It is important to note that we used an available blue LED package, allowing only small currents up to 20 mA to be applied. However, a higher-power excitation light source could achieve higher NIR output power. Photographs obtained under VIS light and pc-NIR-LED light are presented in Figure 6c and d, respectively. Figure 6c shows that the fabricated pc-LED emits NIR light, as demonstrated by the 800 nm long-pass filter. When the pc-LED is turned off, the light does not pass through the filter, hindering visibility behind the setup. However, upon switching on the pc-LED, NIR light passes through the filter, revealing what is behind the setup. Figure 6d shows a conventional color photo illuminated by VIS light, while Figure 6e depicts a black-and-white photo captured under the same illumination. Turning off the VIS light and turning on the NIR-pc-LED, as well as using a NIR camera, enables the capture of black-and-white images (Figure 6f).

The potential application as a UV detector was also investigated for the same sample. Figure 6g illustrates the schematic of the fabricated UV photodetector, where a voltage of 20 V is applied to the sample. A photocurrent is observed when UV light from a 290 nm diode (20 mA) is switched on, as demonstrated in Figure 6h. The photocurrent builds up relatively quickly, followed by a slight increase before stabilizing (bottom panel). A persistent photocurrent is observed after switching off the UV light, reproducible with each consecutive pulse. To mitigate the charging and discharging process associated with this behavior, modulation was implemented using a lock-in amplifier, as described previously. As shown in Figure 6h (upper panel), this approach minimizes the effects related to the persistent photocurrent, and the process becomes reproducible with each pulse, highlighting the utility of this method. Furthermore, Figure 6i depicts the photocurrent dependence on the UV LED current. The UV pulse was gradually increased from 2 to 20 mA, resulting in a linear increase in photocurrent with the increasing UV light intensity.

These findings underscore the promising potential of the Ga_2–*x*_In_*x*_O_3_:Cr^3+^ material in both NIR-pc-LED and UV photodetector applications, demonstrating its versatility and effectiveness across different optoelectronic devices.

## Conclusions

A series of Ga_1.98–*x*_In_*x*_O_3_:0.02Cr^3+^ materials with varying *x* values, from 0.0 to 1.0, were synthesized, exhibiting broadband near-infrared emission. Under 442 nm excitation, Ga_1.98–*x*_In_*x*_O_3_:0.02Cr^3+^ demonstrated a tunable ultrabroadband NIR emission spanning 650 to 1100 nm, primarily from Cr^3+^ ions. The absorption spectra revealed a UV band corresponding to the band-to-band absorption at 260 nm, the charge transfer transition at 300 nm, and two distinct bands in the visible region at 440 and 610 nm. This visible bands are attributed to the ^4^A_2_ → ^4^T_1_ and ^4^A_2_ → ^4^T_2_ transitions of Cr^3+^ ions, respectively. Based on configuration diagrams and estimated activation energies of luminescence quenching, it was determined that nonradiative processes did not result directly from thermally induced relaxation from the excited state ^4^T_2_ to the ground state ^4^A_2_. Photocurrent excitation spectra showed behavior consistent with the absorption spectra, promoting holes into the VB, indicating that photocurrent in these materials involves Cr^3+^ dopants. Notably, a decrease in photoluminescence intensity was observed alongside a monotonically increasing photocurrent with rising temperature, demonstrating the role of thermal ionization in the quenching process of Cr^3+^ in these materials. This study provided the first evidence of luminescence quenching via the hole-creating process in Cr^3+^-doped oxides, suggesting potential applicability to other transition metal ions in dielectric materials.

Finally, the potential of Ga_1.78_In_0.2_O_3_:0.02Cr^3+^ as a dual-purpose material was demonstrated, highlighting its suitability as an emitter for NIR-pc-LEDs and UV detectors. This dual functionality underscores the versatility and practicality of this material in optoelectronic devices for both near-infrared and ultraviolet applications.

## Experimental Section

Gallium oxide (Ga_2_O_3_, 99.99%), indium oxide (In_2_O_3_, 99.99%), and chromium oxide (Cr_2_O_3_, 99.99%) were purchased from Gredmann. To synthesize the Ga_1.98–*x*_In_*x*_O_3_:0.02Cr^3+^, all the precursors were weighed in the stoichiometric ratio and mixed using the agate mortar for 30 min. The mixing powder was transferred into alumina crucibles and put in a muffle furnace. All the samples were heated to 1400 °C for 5 h in an air atmosphere with a heating and cooling rate of 5 °C/min. After the furnace was cooled to room temperature, the samples were again grounded with the agate mortar, and the final products could be obtained.

## Supporting Information Available

Characterization methods, supplementary tables and figures (PDF)
