# Supplementary material for: Photoelectric Studies as the Key to Understanding the Nonradiative Processes in Chromium Activated NIR Materials
Source: J Am Chem Soc. 2024 Aug 2;146(32):22807–17. doi: 10.1021/jacs.4c08011 (PMC11328124; doi:10.1021/jacs.4c08011)
Supplement: Supplementary file 1 — ja4c08011_si_001.pdf [file ja4c08011_si_001.pdf]

Supporting Information

Photoelectric Studies as the Key to Understanding the Non-Radiative Processes in Chromium Activated NIR Materials

Natalia Majewska,$^{\Psi}$ Mu-Huai Fang† and Sebastian Mahlik,$^{\Psi,*}$

$^{\Psi}$Institute of Experimental Physics, Faculty of Mathematics, Physics and Informatics, University of Gdansk, Wita Stwosza 57, 80-308 Gdansk, Poland

†Research Center for Applied Sciences, Academia Sinica, Taipei 11529, Taiwan

EXPERIMENTAL

Characterization

In-house powder X-ray diffraction patterns were characterized by a D8 ADVANCE ECO diffractometer (Bruker) with Cu radiation source (λ ~ 1.54059 Å). Synchrotron Powder X-ray diffraction patterns are collected at the National Synchrotron Radiation Research Center (NSRRC, Taiwan) with the beamline of TPS-19A1 at room temperature (RT) with the wavelength of 0.61992 Å. We used the Total Pattern Analysis Solutions software (TOPAS 6.0) to conduct the Rietveld refinement.

The photoluminescence excitation (PLE) spectra were obtained using a custom setup that included an EQ99X laser-driven Xenon light source (Energetiq) connected to a custom-made grating monochromator, operating within the 250–1000 nm range for excitation. The luminescence was detected using an Andor SR-750-D1 spectrometer equipped with a CCD camera (DU420A-OE), covering the wavelength range of 200–1000 nm. The excitation source was a Kimmon Koha He-Cd laser emitting at 442 nm and an LED with 455 nm wavelengths. All luminescence spectra have been corrected for the apparatus response. The reference spectra were measured using an Optronic Laboratories Calibration Standard Lamp OL 220 with tungsten coiled-coil filaments, enabling spectral irradiance calibrations over the 250 to 2500 nm wavelength range. The spectra obtained from the Andor spectrometer of the Standard Lamp were divided by the Table Spectral Irradiance to obtain the reference curve. Subsequently, the measured luminescence spectra were corrected using this reference. The Standard Lamp was powered by a SCIENCETECH power supply set to 6.5 A.The absorption spectra were acquired using a Quantum Yield Spectrometer C11347 (Hamamatsu) based on diffuse reflectance measurements.

The photocurrent excitation (PCE) spectral measurements were conducted using a custom-made setup comprising a 150 W xenon lamp (LOT Quantum Design) coupled to a grating monochromator (Omni-λ 1509), which operated in the spectral range of 250–1000 nm as the excitation source. The photocurrent was measured using a digital electrometer (Keysight B2987A). The excitation light was modulated at 5 Hz using an optical chopper to enhance the signal-to-noise ratio, and the photocurrent signal was extracted using a lock-in amplifier (Signal Recovery 7270, Ametek Scientific Instruments).

The decay profiles were measured using a setup designed for time-resolved spectroscopy, featuring a PG 401/SH optical parametric generator pumped by a PL2251A pulsed YAG:Nd laser (EKSPLA) as the excitation source. For detection, a 2501S grating spectrometer (Bruker Optics) was used in conjunction with a C4334-01 streak camera (Hamamatsu). The data were recorded as streak images on a 640 by 480 pixel CCD array. The analysis involved software employing a photon counting algorithm, which transformed the recorded data into a 2D matrix representing photon counts plotted against both wavelength and time (streak image). This comprehensive setup facilitated detailed investigation and analysis of the decay dynamics in the spectroscopic measurements.[1]

For temperature-dependent photoluminescence (PL) experiments, temperature control was achieved using the THMSG600 temperature controller integrated with the Linkam stage and the LNP95 liquid nitrogen cooling pump system, enabling precise temperature regulation within the range of 77-600 K. During decay profile measurements, samples were cooled using an APD Cryogenics closed-cycle DE-202 optical cryostat, which provided temperature control ranging from 10 to 450 K. Similarly, for photocurrent excitation (PCE) measurements, sample cooling was facilitated by a custom-made cryogenics closed-cycle optical cryostat, allowing temperature adjustment within the range of 10 to 300 K.

RESULTS

Structural Analysis

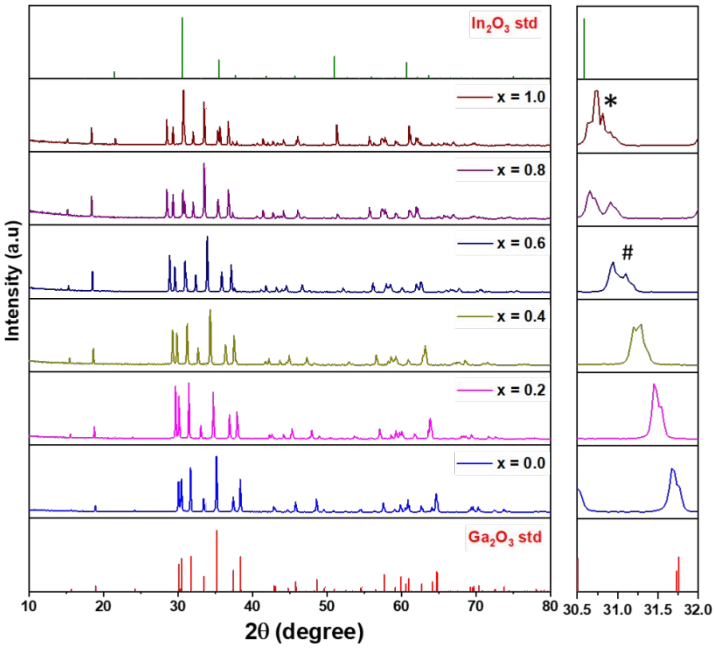

Figure S1. In-house XRD of GIOC (x = 0.0–1.0) with $Cu_{K\alpha}$ radiation source. The asterisk and pound sign indicate the undistinguishable diffraction peaks.

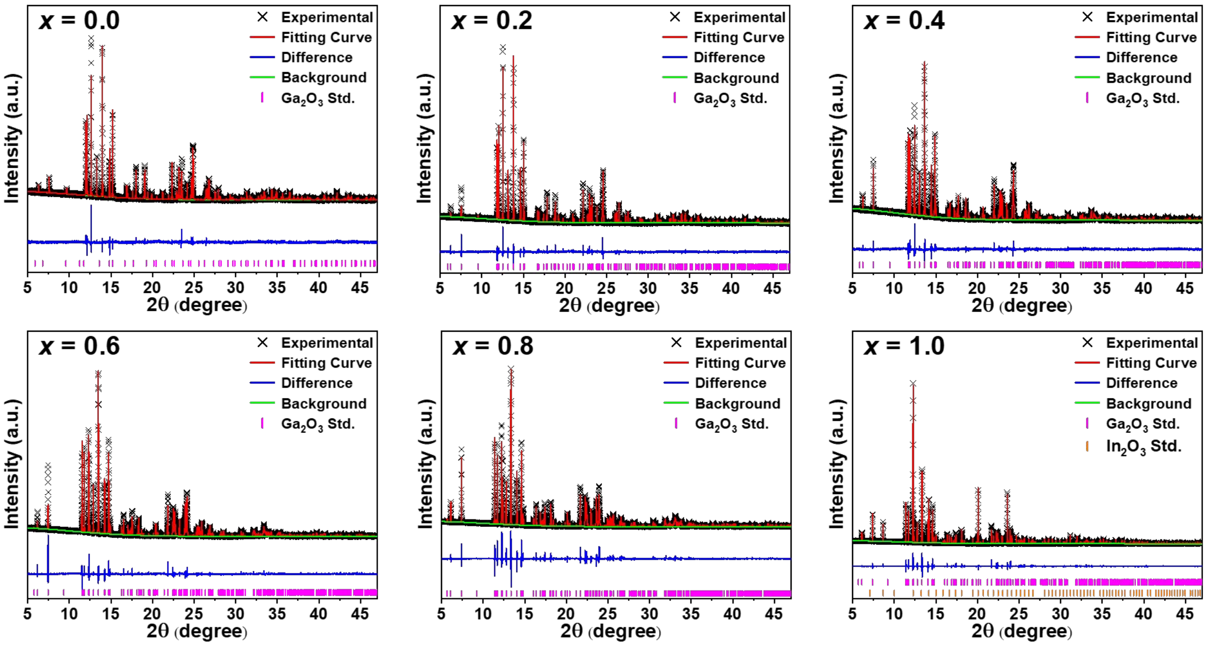

Figure S2. Rietveld refinement of GIOC (x = 0.0–1.0).

Table S1. Atomic positions, occupancies, and atomic displacement parameters of GIOC with x = 0.

$x = 0.0$

Site	*Np*	*x*	*y*	*z*	*Occ*	*Beq*
Ga1	4	0.0902(1)	0	0.7938(3)	1	0.18(2)
Ga2	4	0.3415(1)	0	0.6853(2)	1	0.18(2)
O1	4	0.1580(5)	0	0.111(1)	1	0.05(7)
O2	4	0.4975(5)	0	0.258(1)	1	0.05(7)
O3	4	0.8303(5)	0	0.426(1)	1	0.05(7)

Table S2. Atomic positions, occupancies, and atomic displacement parameters of GIOC with *x* = 0.2.

x = 0.2

Site	*Np*	*x*	*y*	*z*	*Occ*	*Beq*
Ga1	4	0.0906(1)	0	0.7932(2)	0.7932(2)	0.38(1)
In1	4	0.0906(1)	0	0.7932(2)	0.017(3)	0.38(1)
Ga2	4	0.34283(7)	0	0.6876(2)	0.6876(2)	0.38(1)
In2	4	0.34283(7)	0	0.6876(2)	0.183(3)	0.38(1)
O1	4	0.1645(5)	0	0.110(1)	0.110(1)	0.69(6)
O2	4	0.4902(4)	0	0.2556(9)	0.2556(9)	0.69(6)
O3	4	0.8255(4)	0	0.436(1)	0.436(1)	0.69(6)

Table S3. Atomic positions, occupancies, and atomic displacement parameters of GIOC with *x* = 0.4.

x = 0.4

Site	*Np*	*x*	*y*	*z*	*Occ*	*Beq*
Ga1	4	0.0908(1)	0	0.7936(2)	0.999(3)	0.38(1)
In1	4	0.0908(1)	0	0.7936(2)	0.001(3)	0.38(1)
Ga2	4	0.34404(7)	0	0.6882(2)	0.601(3)	0.38(1)
In2	4	0.34404(7)	0	0.6882(2)	0.399(3)	0.38(1)
O1	4	0.1599(4)	0	0.094(1)	1	0.68(7)
O2	4	0.4884(4)	0	0.2468(8)	1	0.68(7)
O3	4	0.8238(4)	0	0.4348(9)	1	0.68(7)

Table S4. Atomic positions, occupancies, and atomic displacement parameters of GIOC with *x* = 0.6.

x = 0.6

Site	*Np*	*x*	*y*	*z*	*Occ*	*Beq*
Ga1	4	0.09078(8)	0	0.7915(2)	0.990(3)	0.37(1)
In1	4	0.09078(8)	0	0.7915(2)	0.010(3)	0.37(1)
Ga2	4	0.34498(6)	0	0.6884(1)	0.410(3)	0.37(1)
In2	4	0.34498(6)	0	0.6884(1)	0.590(3)	0.37(1)
O1	4	0.1604(4)	0	0.0816(8)	1	0.54(6)
O2	4	0.4869(3)	0	0.2531(8)	1	0.54(6)
O3	4	0.8246(3)	0	0.4311(8)	1	0.54(6)

Table S5. Atomic positions, occupancies, and atomic displacement parameters of GIOC with *x* = 0.8.

x = 0.8						
Site	*Np*	*x*	*y*	*z*	*Occ*	*Beq*
Ga1	4	0.08977(9)	0	0.7910(2)	0.999(3)	0.38(1)
In1	4	0.08977(9)	0	0.7910(2)	0.001(3)	0.38(1)
Ga2	4	0.34577(6)	0	0.6887(1)	0.201(3)	0.38(1)
In2	4	0.34577(6)	0	0.6887(1)	0.799(3)	0.38(1)
O1	4	0.1569(5)	0	0.0885(10)	1	0.59(7)
O2	4	0.4827(4)	0	0.2482(9)	1	0.59(7)
O3	4	0.8224(4)	0	0.4326(9)	1	0.59(7)

Table S6. Atomic positions, occupancies, and atomic displacement parameters of GIOC with *x* = 1.0.

x = 1.0 (Ga_2O_3 phase)						
Site	*Np*	*x*	*y*	*z*	*Occ*	*Beq*
Ga1	4	0.08967(9)	0	0.7908(2)	0.87(2)	0.45(1)
In1	4	0.08967(9)	0	0.7908(2)	0.13(2)	0.45(1)
Ga2	4	0.34571(6)	0	0.6887(1)	0.02(2)	0.45(1)
In2	4	0.34571(6)	0	0.6887(1)	0.98(2)	0.45(1)
O1	4	0.1553(5)	0	0.084(1)	1	0.16(9)
O2	4	0.4839(4)	0	0.2529(9)	1	0.16(9)
O3	4	0.8224(4)	0	0.4262(9)	1	0.16(9)

x = 1.0 (In_2O_3 phase)						
Site	*Np*	*x*	*y*	*z*	*Occ*	*Beq*
In1	8	0.25	0.25	0.25	0.77(4)	0.34(1)
Ga1	8	0.25	0.25	0.25	0.23(4)	0.34(1)
In2	24	0.46658(8)	0	0.25	0.85(4)	0.34(1)
Ga2	24	0.46658(8)	0	0.25	0.15(4)	0.34(1)
O1	48	0.3909(8)	0.1585(7)	0.3823(8)	1	1.2(2)

Table S7. Refined parameters of GIOC with x = 0.0–1.0.

x =	0.0	0.2	0.4	0.6	0.8	1.0
Ga_2O_3 phase (wt%)	100	100	100	100	100	75.1(9)
In_2O_3 phase (wt%)	0	0	0	0	0	24.9(9)
a (Å)	12.2263(2)	12.3743(2)	12.5219(2)	12.6594(1)	12.8071(9)	12.8078(1)
b (Å)	3.04019(4)	3.07796(6)	3.11536(5)	3.14964(3)	3.1862(2)	3.18641(3)
c (Å)	5.8076(7)	5.8445(1)	5.8824(1)	5.92076(5)	5.9661(4)	5.96610(4)
β (°)	103.8335(5)	103.5741(4)	103.2397(5)	102.8590(5)	102.4315(5)	102.4190(4)
V (Å^3)	209.606(5)	216.387(7)	223.376(7)	230.154(4)	237.75(3)	237.784(3)
R_{wp} (%)	7.71	7.42	6.46	6.96	10.22	8.76
R_p (%)	5.95	5.68	4.87	4.96	6.83	5.69
GOF	6.23	4.98	4.4	4.78	6.34	3.69

Room Temperature Photoluminescence Analysis

Table S8 displays the energies of the maxima for the $^4A_2 \rightarrow ^4T_1$ and $^4A_2 \rightarrow ^4T_2$ transitions determined from excitation spectra, along with the $^4T_2 \rightarrow ^4A_2$ transition from emission spectra. This information allows us to calculate the crystal field parameter Dq, which describes the interactions between 3d electrons and ligand ions, and the Racah parameter B, representing the interaction between 3d electrons in Cr^{3+}. The energy of the excitation band maximum for the $^4A_2 \rightarrow ^4T_2$ transition is equivalent to the $10Dq$. The Racah parameters B can be calculated using the following equation:[2]

$$B = Dq \frac{\left[\frac{\Delta E}{Dq}\right]^2 - 10 \cdot \frac{\Delta E}{Dq}}{15\left(\frac{\Delta E}{Dq} - 8\right)} (S1)$$

where ΔE is the difference between the energy of the $^4A_2 \rightarrow ^4T_1$ and $^4A_2 \rightarrow ^4T_2$ transitions. The calculated Dq and B parameters are gathered in Table S8. The $S\hbar\omega$ value was estimated using following equation:

$$S\hbar\omega = \frac{E(^4A_2 \rightarrow ^4T_2) - E(^4T_2 \rightarrow ^4A_2)}{2} (S2)$$

where $E(^4A_2\rightarrow{}^4T_2)$ is the energy of $^4A_2\rightarrow{}^4T_2$ transition, taken from the band maksimum in PLE spectra, and $E(^4T_2\rightarrow{}^4A_2)$ is the energy of $^4T_2\rightarrow{}^4A_2$ transition, taken from the band maximum in PL sepctra.

Table S8. Energy of $^4A_2\rightarrow{}^4T_1$ (4F) and $^4A_2\rightarrow{}^4T_2$ (4F) excitation bands and $^4T_2\rightarrow{}^4A_2$ emission band maximum, value of estimated $S\hbar\omega$ and crystal filed and Racah parameters Dq and B

x	$E(^4A_2\rightarrow{}^4T_1)$ (cm^{-1})	$E(^4A_2\rightarrow{}^4T_2)$ (cm^{-1})	$E(^4T_2\rightarrow{}^4A_2)$ (cm^{-1})	$S\hbar\omega$ (cm^{-1})	Dq (cm^{-1})	B (cm^{-1})
**0.0**	22680 ± 340	16470 ± 250	13650 ± 200	1415 ± 20	1647 ± 25	610 ± 9
**0.2**	22270 ± 330	16040 ± 240	12760 ± 190	1639 ± 25	1604 ± 24	618 ± 9
**0.4**	21950 ± 330	15510 ± 230	12240 ± 180	1637 ± 25	1551 ± 23	650 ± 10
**0.6**	21390 ± 320	15220 ± 230	11960 ± 180	1628 ± 27	1522 ± 23	620 ± 10
**0.8**	21230 ±320	14900 ± 220	11600 ± 170	1654 ± 25	1490 ± 22	650 ± 10

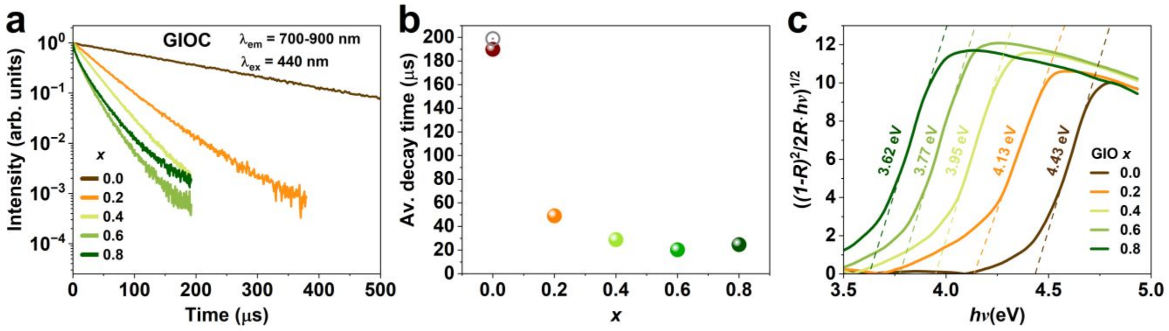

Figure S3. RT x dependence of (a) decay profiles and (b) the calculated average decay times of GIOC of x = 0.0 - 0.8. The decay times taken from the single-exponential fitting are given for x = 0.0 (grey circle). (c) Optical band gap determination for indirect bandgap.

Temperature-Dependent Photoluminescence and Photocurrent Analysis

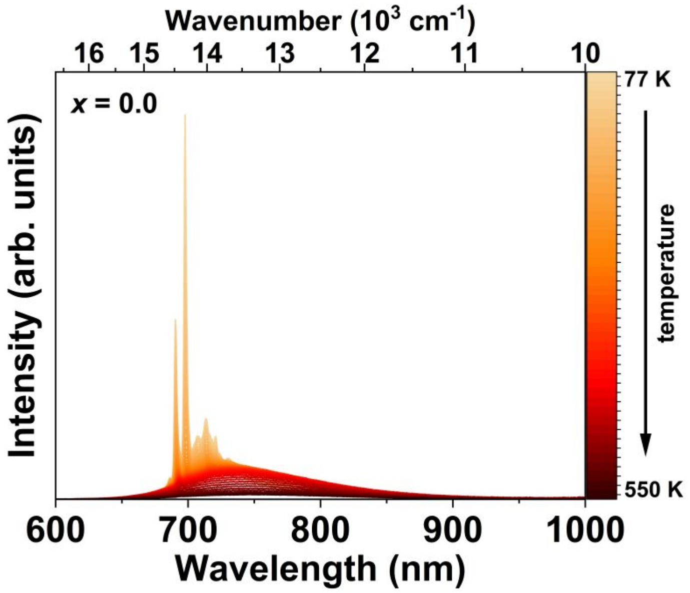

Figure S4. Temperature-dependent emission spectra of GIOC x = 0.0 upon excitation at 455 nm.

Figures S5a and b illustrate the temperature dependence of luminescence decays for GIOC samples with x = 0.2 and 0.6. The decays exhibit a multi-exponential nature due to the distribution of crystal field strengths around Cr^{3+} in mixed ion samples. Figure S5c presents the calculated decay times as a function of temperature for x = 0.2 and 0.6. Due to the multi-exponential decay, the average decay time was computed, eq. (1).

Typically, for Cr^{3+} ions in a strong crystal field at 10 K, the radiative lifetime values remain unaffected by the thermal occupation of the $^4T_2$ state, thus representing the radiative lifetime of the $^2E \rightarrow {}^4A_2$ transition. However, the transition probability of $^2E \rightarrow {}^4A_2$ is influenced by the presence of the $^4T_2$ state due to spin-orbit interaction.[3] It is noteworthy that the lifetime at 10 K is not solely the radiative lifetime of the $^2E \rightarrow {}^4A_2$ transition but is affected by a faster $^4T_2 \rightarrow {}^4A_2$ transition, owing to the distributions of the Cr^{3+} local environment and crystal field, resulting in broadband emission with faster decay even at 10 K. With increasing x, the contribution of the $^4T_2 \rightarrow {}^4A_2$ transition increases. Consequently, the lifetime at 10 K varies significantly, from 88 µs for x = 0.2 to 29 µs for x = 0.6. In the case of x = 0.2 (where line emission is also observed at 100 K), two temperature regions exhibit decreasing decay times. In the low-temperature region (10–200 K), the decrease in decay times is accompanied by an increase in the intensity of the $^4T_2 \rightarrow {}^4A_2$ broadband emission with a shorter decay time compared to the $^2E \rightarrow {}^4A_2$ line emission. In the high-temperature region (>300 K), the decrease in decay times is attributed to

luminescence quenching. For x = 0.6, the emission consists solely of broadband related to the $^4T_2 \rightarrow ^4A_2$ transition, and the lifetime remains stable up to 200 K, equaling the decay time of the $^4T_2 \rightarrow ^4A_2$ broadband emission. The decrease in decay times at higher temperatures is again related to luminescence quenching.

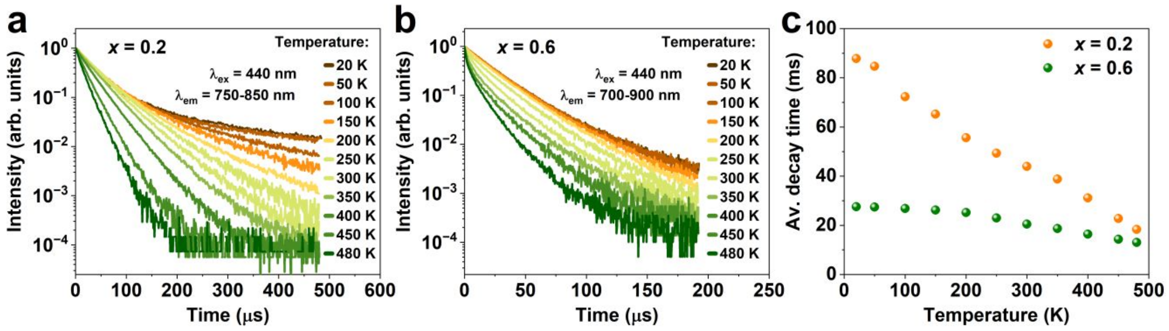

Figure S5. Temperature-dependent decay profiles of GIOC (a) x = 0.2 and (b) x = 0.6 upon excitation at 440 nm. (c) Calculated average decay times.

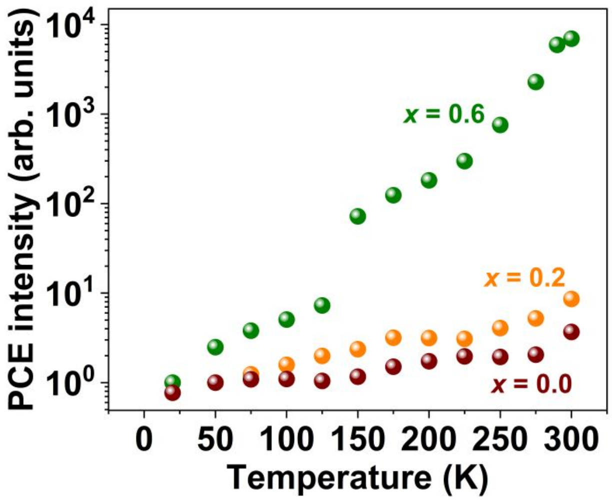

Figure S6. Integrated PCE intensity of GIOC, x = 0.0, 0.2, 0.6.

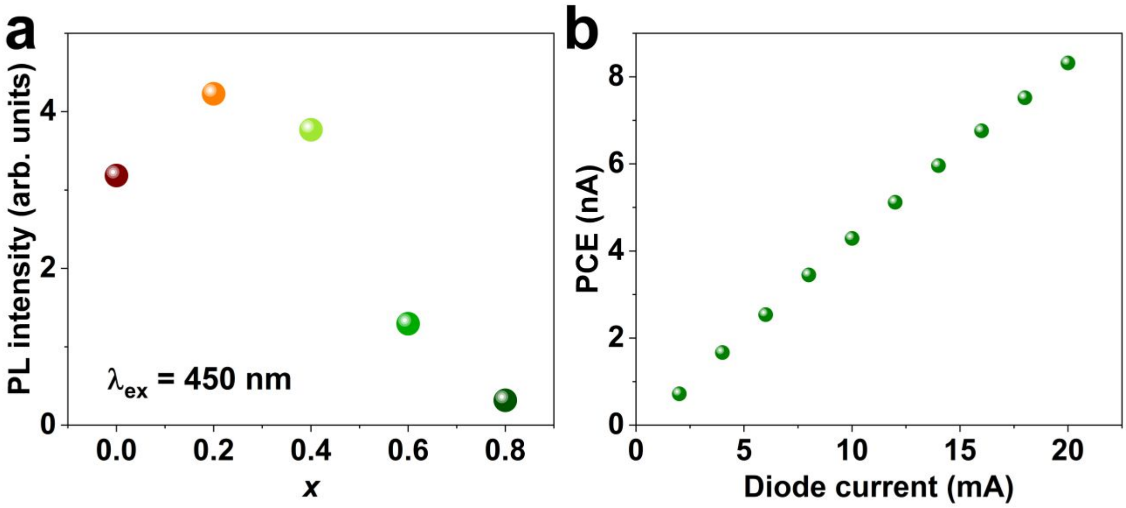

Figure S7. (a) The *x*- dependent emission intensity of GIOC $x = 0.0–0.8$ upon excitation at 450 nm. PCE dependence of excitation source UV-LED current.

REFERENCES

(1) Kubicki, A. A.; Bojarski, P.; Grinberg, M.; Sadownik, M.; Kukliński, B. Time-Resolved Streak Camera System with Solid State Laser and Optical Parametric Generator in Different Spectroscopic Applications. *Opt. Commun.* **2006**, *263* (2), 275–280..

(2) Henderson, B.; Imbusch, G. F. *Optical Spectroscopy of Inorganic Solids*; Monographs on the Physics and Chemistry of Materials; Oxford University Press: Oxford, New York, 2006.

(3) Grinberg, M. $^2E \rightarrow {}^4A_2$ Fluorescence of Cr^{3+} in High and Intermediate Field Garnets. *J. Lumin.* **1993**, *54* (6), 369–382.
